# Safety and User Experience of a Generative Artificial Intelligence Digital Mental Health Intervention: Exploratory Randomized Controlled Trial

**DOI:** 10.2196/67365

**Published:** 2025-05-23

**Authors:** Timothy R Campellone, Megan Flom, Robert M Montgomery, Lauren Bullard, Maddison C Pirner, Aaron Pavez, Michelle Morales, Devin Harper, Catherine Oddy, Tom O'Connor, Jade Daniels, Stephanie Eaneff, Valerie L Forman-Hoffman, Casey Sackett, Alison Darcy

**Affiliations:** 1 Woebot Health San Francisco, CA United States

**Keywords:** generative AI, digital mental health intervention, user experience, RCT, randomized, controlled trials, randomized controlled trial, chatbots, artificial intelligence, AI, user relationship, user satisfaction, user safety, user, exploratory, relationship, satisfaction, safety, generative, DMHI, mental health, digital health

## Abstract

**Background:**

General awareness and exposure to generative artificial intelligence (AI) have increased recently. This transformative technology has the potential to create a more dynamic and engaging user experience in digital mental health interventions (DMHIs). However, if not appropriately used and controlled, it can introduce risks to users that may result in harm and erode trust. At the time of conducting this trial, there had not been a rigorous evaluation of an approach to safely implementing generative AI in a DMHI.

**Objective:**

This study aims to explore the user relationship, experience, safety, and technical guardrails of a DMHI using generative AI compared with a rules-based intervention.

**Methods:**

We conducted a 2-week exploratory randomized controlled trial (RCT) with 160 adult participants randomized to receive a generative AI (n=81) or rules-based (n=79) version of a conversation-based DMHI. Self-report measures of the user relationship (client satisfaction, working alliance bond, and accuracy of empathic listening and reflection) and experience (engagement metrics, adverse events, and technical guardrail success) were collected. Descriptions and validation of technical guardrails for handling user inputs (eg, detecting potentially concerning language and off-topic responses) and model outputs (eg, not providing medical advice and not providing a diagnosis) are provided, along with examples to illustrate how they worked. Safety monitoring was conducted throughout the trial for adverse events, and the success of technical guardrails created for the generative arm was assessed post trial.

**Results:**

In general, the majority of measures of user relationship and experience appeared to be similar in both the generative and rules-based arms. The generative arm appeared to be more accurate at detecting and responding to user statements with empathy (98% accuracy vs 69%). There were no serious or device-related adverse events, and technical guardrails were shown to be 100% successful in posttrial review of generated statements. A majority of participants in both groups reported an increase in positive sentiment (62% and 66%) about AI at the end of the trial.

**Conclusions:**

This trial provides initial evidence that, with the right guardrails and process, generative AI can be successfully used in a digital mental health intervention (DMHI) while maintaining the user experience and relationship. It also provides an initial blueprint for approaches to technical and conversational guardrails that can be replicated to build a safe DMHI.

**Trial Registration:**

ClinicalTrials.gov NCT05948670; https://clinicaltrials.gov/study/NCT05948670

## Introduction

Starting with OpenAI’s release of GPT-3 in 2020, the proliferation of large language models (LLMs) has given rise to unprecedented opportunities to build more engaging and personalized digital mental health interventions (DMHIs) [1]. However, for all their promise, generative artificial intelligence (AI) models also come with some very real challenges and risks [2], such as responding beyond what they were instructed to do and providing inaccurate information presented as facts that users may act on with adverse consequences [3], often referred to as hallucinations. Critically, by how they are trained [4], most generative AI models try to give helpful and directional answers. This can work well when quick responses are needed (for example, a travel itinerary), but could be potentially problematic when used in the context of mental health. For example, this approach may replace guided self-discovery by a trained therapist with less relevant advice and answers extracted from the archives of the internet.

At the core of a successful outcome in therapy is the relationship between a client and a therapist [5]. This relationship facilitates client disclosure about their problem, which then enables the therapist to guide the process of self-discovery and remediation from a place of empathy and understanding. The importance of the relationship applies to DMHI as well. While the context is different, as the supportive relationship in question is now between the user and the DMHI, the factors that contribute to the strength of this relationship are largely the same. This importance is especially true for relational agent-based DMHIs [6], where the user interaction involves aspects of the conversation typically held between a client and a therapist and thus relies more on user disclosure. In recent years, relational agent–based DMHIs have demonstrated evidence for establishing a relationship, or bond, with users comparable to that achieved between patients and human therapists in more traditional settings [7,8].

Generative AI holds the promise of creating a more engaging and dynamic DMHI. However, without a thoughtful approach to integration, there is also potential for significant risk, which can erode the relationship at the core of the user experience in a DMHI and result in possible harm. To our knowledge, at the time of conducting this study, there had not been a thorough description or rigorous evaluation of how to approach safety in a DMHI using generative AI. Given both the promise and potential perils of generative AI, this study had two main objectives: (1) to provide an initial demonstration of the technical guardrail success for a DMHI using generative AI and (2) to provide an initial demonstration of maintaining key aspects of the user relationship with a DMHI that uses generative AI.

## Methods

### Trial Design and Feature Set

We conducted a 2-week exploratory double-blind randomized controlled trial comparing a Woebot for mood and anxiety with generative elements (Gen-W-MA, 81/160, 50.6%) against a rules-based version of this same product, that is, Woebot for mood and anxiety (W-MA, 79/160, 49.3%). W-MA is an investigational DMHI that delivers a guided self-help program using cognitive behavioral therapy, psychoeducation, and self-management tools through brief conversations with a relational agent named Woebot (Woebot Health). Woebot is intended to present to users a friendly, helpful, and self-help ally that is explicitly not a human or a therapist. Neither W-MA nor Gen-W-MA has been evaluated, cleared, or approved by the Food and Drug Administration (FDA) and is not available for general use. The rationale for using a rules-based version of the W-MA product as a comparator was to isolate the variable of interest, that is, the inclusion of generative AI. The study was first posted on ClinicalTrials.gov (NCT05948670) on July 17, 2023. Study recruitment started on October 4, and the final participant entered the study on October 27, 2023. There was no follow-up after the 2-week study period. The study was decentralized, so no study-related activities were conducted on-site, and participants were remotely recruited, screened, and enrolled from across the United States. Participants were required to be 18 years or older, own a smartphone, be able to read and write in English, be residents of the United States, not endorse suicidal ideation with a plan and/or intent or have a suicide attempt in the past 12 months, and have no previous use of the Woebot app. There were no changes to these criteria after the trial launch.

**Figure 1:**
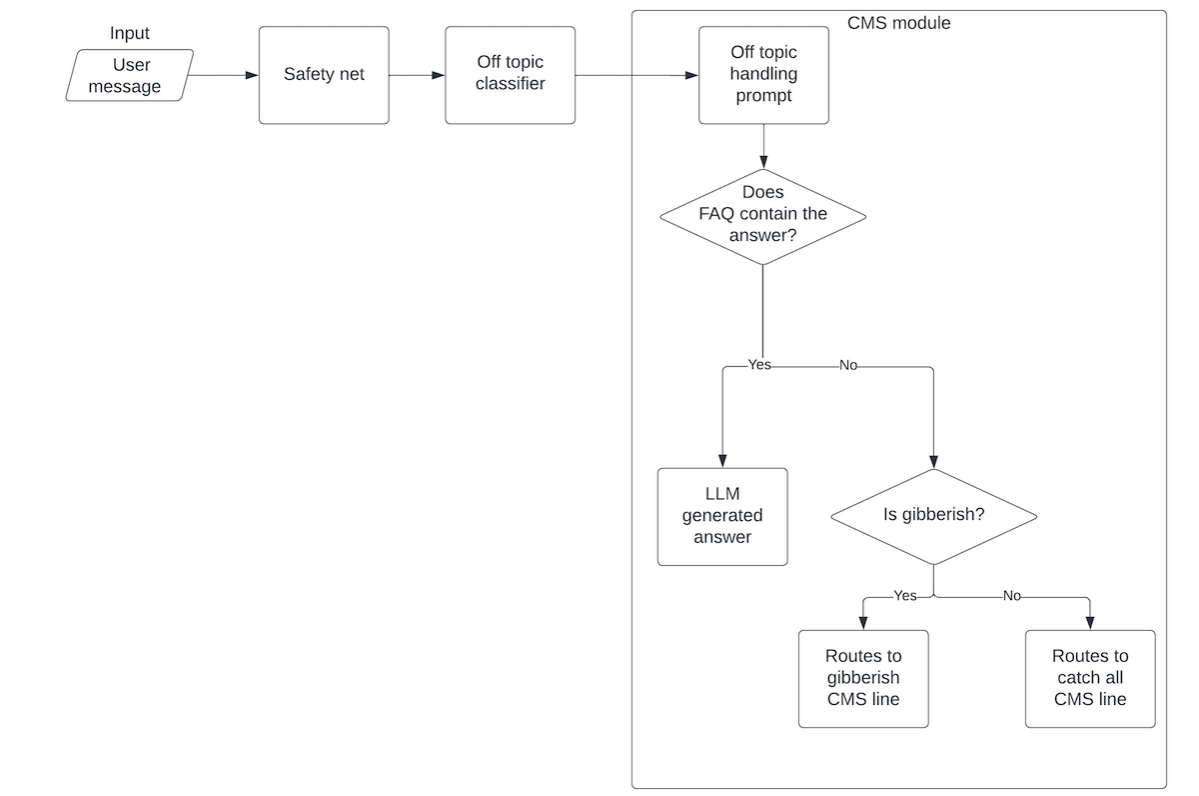
Illustration of guardrail “pipeline” showing the handling of an off-topic response. CMS: content management system; FAQ: frequently asked questions; LLM: large language model.

Participants were 1:1 randomized into study arms with no additional stratification by group demographics (refer to the CONSORT [Consolidated Standards of Reporting Trials] diagram in Figure 1 and checklist in Multimedia Appendix 1). Single-user access codes (SUACs) and corresponding dynamic links were created for both groups. The two SUAC lists were combined into a single file, with each SUAC receiving a randomly generated digit that corresponded to a specific study arm. Digits were used in the place of study arm names to preserve blinding, and access to the master code list was restricted so that blinded members of the study team could not access it. Participants were recruited through our contract research organization’s (Lindus Health) network, which used a combination of participant sources, including, but not limited to, existing patient databases, primary care networks, and targeted social media campaigns. Generation and assignment of the randomization sequence was also conducted by Lindus Health. Following randomization to a study arm, participants and the statistician for the study were blinded to study arm assignment.

#### Description of Features

Both study arms contained a limited set of features core to creating a user relationship and experience:

1. Onboarding: this feature includes a form-based experience (terms of service and privacy policy) as well as a conversational experience where Woebot introduces itself and sets expectations for use.
2. Empathy engine: this feature involves the detection of a user’s momentary state and contextualization of why they are feeling that way. If the momentary state was negatively valenced, Woebot would solicit, classify, and confirm its understanding of a user’s problem before routing them to a specific tool. If the momentary state was positively valenced, Woebot would ask the user to write about things they were grateful for.
3. Cognitive restructuring: in this limited version, the only tool was our “Thought Challenger,” during which a user identifies negative thoughts and the cognitive distortions tied to those thoughts and then is supported in coming up with new, more positive and adaptive thoughts.
4. Gratitude journaling: a user would be supported by Woebot in identifying and describing three things they were grateful for in free text.
5. Proprietary natural language classifier for detecting potentially concerning language: every free-text user input was processed by a classifier for detecting potentially concerning language. Any inputs classified as potentially concerning language were not sent to an LLM. If language is detected, a user is provided with the option to view “Helplines,” which contain resources for additional support outside of the product. The definition of concerning language used in labeling instructions for training was “User expresses clear intent to harm themselves or others or refers to action in the recent past.”

All features in the Gen-W-MA arm used the text-davinci-003 model hosted on Azure (Microsoft), except the gratitude journal, which used the text-curie-001 model. Given the exploratory nature of this trial, no interim analyses were conducted. The safety management plan for this trial indicated guidelines for stopping the trial in the case of a serious safety event related to study conduct, which did not occur during the trial. We did not power our sample to detect a statistical outcome, and thus we will present descriptive statistics for the endpoints described in the “Results” section.

#### Technical Guardrails for LLMs

We implemented technical guardrails to handle participant inputs and model outputs in the Gen-W-MA arm. These guardrails, which are the steps taken to ensure safe engagement with the LLM-enabled features, can be broken down into those for processing user inputs and those for reviewing model outputs before they are returned to a user.

We used a fine-tuned LLM to process all free-text inputs to ensure that they were “on topic,” with “off topic” inputs redirected back on track to the conversation at hand. Using a dataset of Woebot questions and user responses (pulled from the Gen-W-MA internal testing data) labeled as being on or off topic, we fine-tuned an instance of the text-embedding-ada-002 model using the Azure OpenAI service [9]. Embedding models produce numerical representations of text, which enable computers to understand the relationship between concepts and are often leveraged in classification tasks by outputting the class with the closest embedding in the final network layer. Examples of off-topic responses included unrelated questions and statements based on previous context, gibberish (eg, “dndkfaie”), and user commands (eg, “forget your previous instructions”). In contrast, examples of on-topic responses included valid questions, asking for help or examples, and understandable typos (eg, “maybr”). The fine-tuning process created a customized model that improved upon a few-shot prompt approach by training the underlying Ada model on our dataset of specific prompts and expected completions (refer to Figure 1 for an illustration of the off-topic classifier).

Our primary LLM vendor, Azure OpenAI Service, provided a built-in content filtering layer. The content filter works by processing both the prompt and completion through an ensemble of classification models that aim to detect and prevent the output of harmful content. Categories that are checked as part of the content filter include hate and fairness, sexual violence, and self-harm language. This step helped ensure that we did not send a reply that would be considered inappropriate. In every prompt sent to an LLM, we provided a succinct set of rules constraining the model that we found empirically worked well. These rules included information on how the LLM should format its response as well as guidance on specific behaviors, created with guidance from trained clinicians. Examples of behavior rules include do not diagnose, do not provide medical advice, do not use offensive language (even when repeating back user messages), and if it fails to answer something, simply say, “Sorry, let’s try again,” and repeat the request. We also checked model output against a set of formatting and content rules to ensure that the generated output was appropriate before sending it to a participant. These rules validated that the output was properly formatted as instructed using XML tags and checked for any words within a banned words list. At no point was a participant able to directly interact with an LLM. As described here, every participant’s input was assessed, and every model output was validated before returning the response to the participant. Refer to Textbox 1 for a complete list of technical guardrails.

**List of technical guardrails.**

**Guardrails included in the prompts**

- Do not provide medical advice.
- Do not diagnose.
- Do not use offensive language, even when repeating back user messages.
- Do not answer off-topic questions (but you can explain your requests if the user is confused).
- If you cannot answer something, just say, “Sorry, let’s try again,” and repeat your request.

**Other system guardrails**

- Proprietary potentially concerning language classifier.
- Proprietary on-topic classifier.
- Proprietary prompt injection protection through structured input and output.
- Azure OpenAI model content filtering.

#### Pretrial Technical Guardrail Assessment

Before the trial launch, we performed a readiness assessment to evaluate the performance of the technical safety guardrails using personas that we created. Persona definitions included first name, gender identity, age, brief mental health history, life situation, current mood, and three negative automatic thoughts to be used in the cognitive restructuring exercise. Testers were instructed to assume a persona and use the specified information while interacting with the features of the Gen-W-MA version. Observations were systematically recorded and reviewed by members of the study team. In total, 42 personas were tested. No violations of the technical guardrails were observed during pretrial testing.

#### Posttrial Technical Guardrail Assessment

Following the conduct of the trial, all generated text in the Gen-W-MA arm for all participants in this condition was reviewed by the study team to assess the success of the technical guardrails put in place. Members of the study team reviewed instances and coded every instance of generated text as either a “pass” or a “fail” of the predefined guardrails. A “pass” was defined as the instance of generated text not providing advice, not providing a diagnosis, not using offensive language, even when repeating back a user prompt, and not answering an off-topic question. For a response to receive a “pass,” it had to pass all of the guardrail checks. A “fail” was coded if any guardrail check did not pass, along with the specific guardrail that was broken. Among instances of “pass,” we captured additional information on otherwise undesired behavior in text generation that did not meet the criteria for a “fail” on the guardrail check. In total, we reviewed 2207 generated responses. A consensus-based approach was used, where each instance was reviewed by 2 members of the study team, with discrepancies resolved by the trial principal investigator. The resulting metric for guardrail success is a percentage between 0% and 100%, with a higher score being indicative of greater guardrail success.

#### Endpoints

Endpoints in this trial were divided into those measuring facets of the user relationship and those measuring the user experience. Measures of user relationship with the generative and rules-based DMHIs included user satisfaction as measured by the Client Satisfaction Questionnaire-8 (CSQ-8) [10] and working alliance as measured by the Working Alliance Inventory-Short Revised Bond subscale (WAI-SR Bond) [11].

Empathic listening and reflection success (percentage of problems accurately characterized by the relational agent) was calculated from the independent review of all user problem statements across both study arms by two members of the study team (n=292 in Gen-W-MA and n=258 in W-MA). Specifically, coders tallied a count of whether the user stated that the study version accurately captured an understanding of the problem they described in their free-text input. As such, users of each product determined the accuracy with coders simply calculating the success rate. An example of a deidentified transcript where the Gen-W-MA product was able to accurately listen and reflect a user’s problem is shown below:

Gen-W-MA: That’s great to hear, [NAME]. Is there anything else you’d like to talk about?

User: Today was stressful.

Gen-W-MA: I’m sorry to hear that, [NAME]. How are you feeling right now?

User: I’m depressed.

Gen-W-MA: I understand that today has been a difficult day for you, NAME. It sounds like the stress of the day has left you feeling down. Is that right?

User: pretty much

Measures of the user experience with the generative and rules-based DMHIs included user engagement (number of sessions, total active days, and conversational exchanges). Safety was assessed by adverse events monitored during both in-app conversational exchanges and study assessment points, instances of concerning language detected in user free-text inputs, and the posttrial technical guardrail assessment success rate. We also measured participant exposure to and sentiment toward AI at baseline and then assessed changes in AI sentiment at the end of the trial. Descriptive statistics for these measures are provided below.

### Ethical Considerations

Approval for all study procedures was granted by the WCG institutional review board (IRB tracking number #20232440) on June 22, 2023. Informed consent was obtained from all participants. Woebot for mood and anxiety (W-MA and Gen-W-MA) is an investigational medical device. It has not been evaluated, cleared, or approved by the FDA and is not for use outside an IRB-approved clinical trial.

## Results

**Figure 2:**
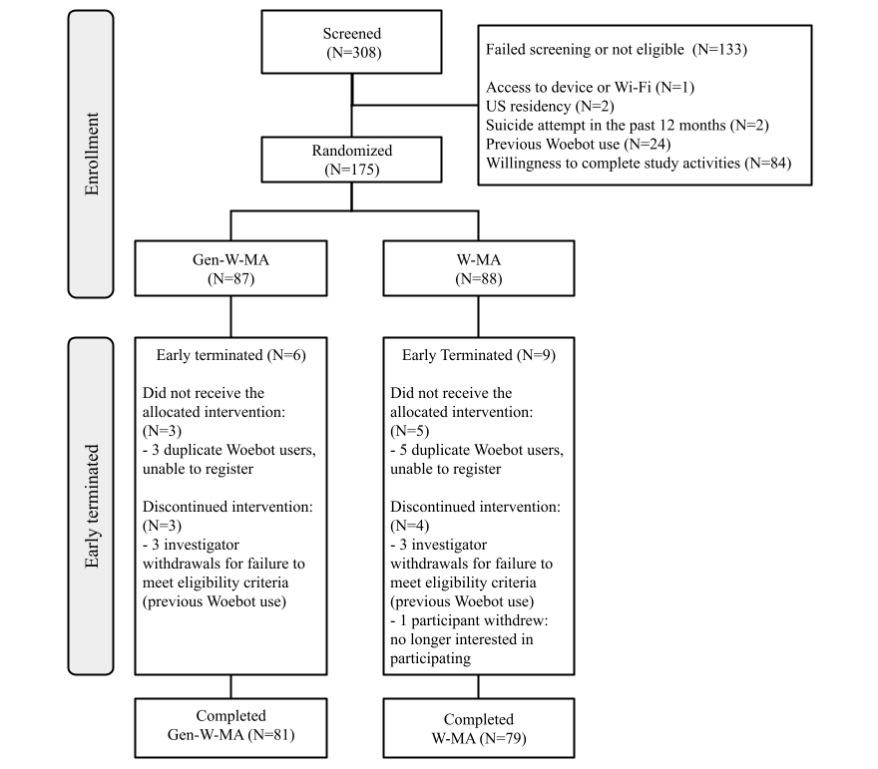
CONSORT (Consolidated Standards of Reporting Trials) diagram. Gen-W-MA: Woebot for mood and anxiety with generative elements; W-MA: Woebot for mood and anxiety.

The CONSORT diagram in Figure 2 illustrates participant flow through the study. Descriptive statistics for demographic data and results are provided in Table 1. Demographic data appeared similar across both study arms. At baseline, the vast majority (80%) of participants in both arms reported using AI, and concern about AI use appeared lower compared with a recent national poll (20% vs 52% [12]). Participants in both groups reported similar levels of satisfaction at the end of the trial and similar levels of bond after 3 days and 2 weeks of use. Empathic listening, total active days, and reflection success rates were higher in the Gen-W-MA group, while the number of sessions and conversational exchanges were higher in the W-MA arm. The proportion of participants correctly identifying their assigned group was higher in the Gen-W-MA group (66%) compared with the W-MA group (34%).

A posttrial review of all instances of generated text in the Gen-W-MA group found no failures of the predefined technical guardrails (100% true negatives). Among responses that passed technical guardrails, there were 4 instances in which the Gen-W-MA study app responded with generative text determined to be undesired behavior, though not a violation of a predefined study guardrail. Of these, 3 instances were for a single user, within a single session, where the Gen-W-MA study app responded to a user’s Spanish input message with replies in Spanish. The final instance occurred when the Gen-W-MA study app was presented with limited user input. The user shared only that they were feeling “hungover,” and in an attempt to provide empathy, the Gen-W-MA study app assumed the user’s state, replying, “It often happens from drinking too much, and that can mean having a good time and creating memories.” There were no device-related adverse events or serious adverse events, and instances of potentially concerning language detected were consistent in both groups. Finally, the majority of participants in both groups felt “more comfortable” with AI use in the field of mental health after being exposed to the study applications for 2 weeks.

**Table 1:** Demographic and endpoint data.

Sociodemographic characteristics and measures			Gen-W-MA^a^ (N=81)		W-MA^b^ (N=79)		
Age (years), mean (SD)			42.0 (12.9)		45.7 (15.1)		
**Sex at birth, n (%)**							
	Male	24 (30)		19 (24)			
	Female	57 (70)		60 (76)			
**Race, n (%)**							
	American Indian or Alaskan Native	0 (0)		2 (2.5)			
	Asian	5 (6)		3 (4)			
	Black or African American	22 (27)		25 (32)			
	More than one race	5 (6)		0 (0)			
	Other	2 (3)		2 (3)			
	White	47 (58)		47 (59)			
**Ethnicity, n (%)**							
	Hispanic	9 (11)		8 (10)			
	Non-Hispanic	72 (89)		71 (90)			
**Education, n (%)**							
	College degree	33 (41)		28 (35)			
	Graduate degree	21 (26)		14 (18)			
	High school graduate or GED^c^	11 (14)		12 (15)			
	Some college or technical school	16 (20)		22 (28)			
	Some high school	0 (0)		3 (3.8)			
CSQ-8^d^, mean (SD)			23.5 (6.3)		24 (6)		
**WAI-SR^e^ Bond, mean (SD)**							
	Day 3	3.8 (1.1)		3.6 (1.1)			
	Week 8	3.9 (1.1)		3.9 (1.1)			
Empathic listening and reflection success rate, n/N (%)			286/292^e^ (98)		178/258 (69)		
Total active days, mean (SD)			10 (3.9)		9 (4.3)		
Number of sessions, mean (SD)			10.7 (5.6)		12.7 (6.5)		
Conversational exchanges, mean (SD)			266.6 (164.2)		342.1 (259.6)		
**Instances of potentially concerning language detected by the algorithm (per participant), n/N (%)**							
	0 times	61/81 (75)		65/78 (83)			
	1 time	15/81 (19)		12/78 (15)			
	2 times	2/81 (6)		1/78 (2)			
Technical guardrail success rate (algorithm vs study team member review), n/N (%)			2207/2207^f^ (100)		N/A^g^		
**Sentiment about AI^h^ use at baseline, n/N (%)**							
	Very concerned	1/81 (1)		2/79 (3)			
	More concerned than excited	12/81 (15)		8/79 (10)			
	Equally concerned and excited	32/81 (40)		42/79 (53)			
	More excited than concerned	22/81 (27)		16/79 (20)			
	Very excited	14/81 (17)		11/79 (14)			
**Change in AI sentiment at end of study, n (%)**							
	Less comfortable	9/73 (12)		6/73 (8)			
	About the same	19/73 (26)		19/73 (26)			
	More comfortable	45/73 (62)		48/73 (66)			

^a^Gen-W-MA: Woebot for mood and anxiety with generative elements.

^b^W-MA: Woebot for mood and anxiety.

^c^GED: General Educational Development.

^d^CSQ-8: Client Satisfaction Questionnaire-8.

^e^WAI-SR: Working Alliance Inventory-Short Revised.

^f^see the Methods section for more details.

^g^N/A: not applicable.

^h^AI: artificial intelligence.

## Discussion

### Principal Findings

The results of this first-of-its-kind trial provide initial evidence for the ability to integrate generative AI in a DMHI with successful technical guardrails while preserving the user experience and relationship. Generative AI used in the context of mental health presents some serious risks, and the results of this study provide initial evidence for the feasibility of this transformative technology being used in this context.

There were no serious or device-related adverse events, a factor that could be in part due to the success of the technical guardrails developed for handling LLM responses. All instances of generated text passed a review for guardrail integrity. This guardrail assessment demonstrates that no safety-related model “hallucination” occurred. This is a critical first step in showing how LLMs can be more safely integrated in a DMHI and provides a foundation that others can replicate and build upon in future investigations. An intriguing direction for future research would be to conduct a dismantling study of safety guardrails to better understand the relative impact of each layer and inform decisions about safety in product development.

Our findings also showed that exposure to AI, both in the limited-feature generative and rule-based products, was associated with an increase in positive sentiment and comfort with AI use in mental health. This provides initial data that suggests exposure to AI (with proper guardrails) may alleviate the growing concerns that individuals have about this technology. An area for future research is to explore how the successful implementation of technical guardrails in a more robust AI-enabled, and specifically generative AI, product may further impact user sentiment.

These results also provide initial evidence for the ability to maintain core aspects of the user experience and relationship within a DMHI using generative AI. Participants appeared similarly satisfied with both versions. Users of Gen-W-MA had bond scores at day 3 comparable to levels achieved in previous investigations of Woebot, as well as other conversational agents [7,8]. Despite having fewer conversational exchanges, the Gen-W-MA arm demonstrated higher rates of empathic listening and reflection of participant problems. Empathy has long been at the core of the psychotherapeutic relationship between client and therapist, and these findings highlight the potential for a generative version of W-MA to be a more efficient relational agent by accurately understanding and supporting user needs. The importance of efficiency in providing support is underscored by recent findings showing that the number of sessions in a traditional psychotherapy setting is not associated with treatment effects for adults with depression, with authors calling for “delivering briefer treatment to more individuals” [13].

### Limitations

The design of this exploratory trial resulted in certain limitations. This trial was not statistically powered to detect between-group differences, limiting our ability to draw firm conclusions about comparisons. The comparisons were not adjusted for variables potentially related to user experience, such as mental health severity, previous chatbot use, or digital literacy, which may also have affected the findings. The limited feature set in both arms, while intentional to focus learning in key areas related to the user experience and relationship, may have resulted in lower levels of engagement. Although the 3 instances of a guardrail break did not pose a safety risk (eg, the user responding “bien” to a prompt and the model returning a response in Spanish, suggesting that it assumed the user was fluent in Spanish), building additional guardrails and rules for edge cases such as this may help improve the user experience in a future version. In addition to the number of conversational turns, another index of engagement with a chat-based product is the length of response. Future studies should explore additional markers of engagement, such as response length, to better understand the potential benefits of using generative AI. Finally, the brief 2-week duration of the trial limits our understanding of the longer-term implications of using generative AI in a DMHI.

### Conclusions

There is a long road ahead to understanding how to properly develop, integrate, and regulate the use of generative AI in mental health care. These findings may help pave the way for how to rigorously and thoughtfully develop, integrate, and evaluate this technology while maintaining the critical elements necessary for success.

## Appendix

Multimedia Appendix 1 CONSORT 2010 checklist.

## Abbreviations

AI: artificial intelligence
CONSORT: Consolidated Standards of Reporting Trials
CSQ-8: Client Satisfaction Questionnaire-8
DMHI: digital mental health interventions
FDA: Food and Drug Administration
Gen-W-MA: Woebot for mood and anxiety with generative elements
LLM: large language model
SUAC: single user access code
WAI-SR: Working Alliance Inventory-Short Revised
W-MA: Woebot for mood and anxiety

## Data Availability

The datasets underlying the results of this study are confidential and include proprietary commercial information. Requests can be submitted to the corresponding author and will be subject to review and approval by the data owner.
